# Orally Administrated Cinnamon Extract Reduces β-Amyloid Oligomerization and Corrects Cognitive Impairment in Alzheimer's Disease Animal Models

**DOI:** 10.1371/journal.pone.0016564

**Published:** 2011-01-28

**Authors:** Anat Frydman-Marom, Aviad Levin, Dorit Farfara, Tali Benromano, Roni Scherzer-Attali, Sivan Peled, Robert Vassar, Daniel Segal, Ehud Gazit, Dan Frenkel, Michael Ovadia

**Affiliations:** 1 Department of Molecular Microbiology and Biotechnology, Tel Aviv University, Tel Aviv, Israel; 2 Department of Zoology, Tel Aviv University, Tel Aviv, Israel; 3 Department of Neurobiology, Tel Aviv University, Tel Aviv, Israel; 4 Department of Cell and Molecular Biology, Northwestern University, Chicago, Illinois, United States of America; Johns Hopkins, United States of America

## Abstract

An increasing body of evidence indicates that accumulation of soluble oligomeric assemblies of β-amyloid polypeptide (Aβ) play a key role in Alzheimer's disease (AD) pathology. Specifically, 56 kDa oligomeric species were shown to be correlated with impaired cognitive function in AD model mice. Several reports have documented the inhibition of Aβ plaque formation by compounds from natural sources. Yet, evidence for the ability of common edible elements to modulate Aβ oligomerization remains an unmet challenge. Here we identify a natural substance, based on cinnamon extract (CEppt), which markedly inhibits the formation of toxic Aβ oligomers and prevents the toxicity of Aβ on neuronal PC12 cells. When administered to an AD fly model, CEppt rectified their reduced longevity, fully recovered their locomotion defects and totally abolished tetrameric species of Aβ in their brain. Furthermore, oral administration of CEppt to an aggressive AD transgenic mice model led to marked decrease in 56 kDa Aβ oligomers, reduction of plaques and improvement in cognitive behavior. Our results present a novel prophylactic approach for inhibition of toxic oligomeric Aβ species formation in AD through the utilization of a compound that is currently in use in human diet.

## Introduction

Alzheimer's disease (AD) is a progressive, irreversible brain disorder with an unclear etiology and no cure. Symptoms include memory loss, confusion, impaired judgment, disorientation, and loss of language skills [1]. In the past two decades, a large number of experimental studies have established a pathological role for Aβ in AD [1]–[2]. However, recent debate has focused on whether Aβ amyloid fibrils or Aβ soluble oligomers are the main neurotoxic species which contribute to neurodegeneration and dementia. Considerable evidence has indicated that amyloid fibrils are toxic [1]. Yet, recent studies support the notion that it is actually the early soluble oligomers that are the primary neurotoxic agents [3]–[9]. In particular, 56 kDa (56*) dodecameric oligomers of Aβ were shown to correlated with the deterioration of cognitive functions in AD model mice and their reintroduction into normal brains resulted in memory impairment [7], [10].

Despite a significant increase in our understanding of the pathogenesis of AD, therapeutic options are still very limited and aim only at amelioration of symptoms. More recent therapeutic approaches aim at removing aggregated Aβ and decreasing the production of the pathogenic Aβ_42_ peptide [11]. Yet, novel observations suggest that the endogenous Aβ peptides may normally have a crucial role in activity-dependent regulation of synaptic vesicle release [12], hence methods used to abolish all Aβ production may in fact aggravate synapse loss in Alzheimer's disease. Therefore, it may be advantageous to target the earliest stages of Aβ oligomerization, thus removing all potential toxic species of Aβ. Indeed, pervious work by our group and others has already demonstrated that targeting the early process of Aβ molecular recognition is a very promising approach for the treatment of AD [13]–[16].

Plants have a long history as a rich source of new bioactive compounds for drug discovery and may have advantages in relation to efficacy. Several reports documented the effectiveness of herbal extracts over isolated material, in protection against lipid peroxidation [17] and anti cancer effects [18]. For example, a mixture of carotenoids have been found to be more effective than any one single carotenoid in protecting liposomes against lipid peroxidation [17].

Recent studies have shown inhibition of Aβ plaque formation *in vitro* and *in vivo* by compounds from natural sources [19]–[21]. Still, evidence for the capability of common edible elements to inhibit Aβ oligomerization *in vivo* remains a challenge. Cinnamon is widely used by humans, both as a spice and as a traditional medicine. It is, perhaps, one of the oldest herbal medicines, having been mentioned in the Bible (*Exodus, Proverbs* and *Song of Songs*) and in Chinese texts as long as 4,000 years ago [22]. The unique healing abilities of cinnamon are due to various components such as cinnamaldehyde, eugenol, cinnamyl acetate, and cinnamyl alcohol, in addition to a wide range of other volatile substances including safrole, coumarin and cinnamic acid esters [22]. Cinnamon has unique medicinal abilities such as blood sugar control [23], anti-oxidant [22], anti inflammatory [24] and anti-microbial activities [25]. Furthermore, it was demonstrated that cinnamon has an inhibitory effect on Tau aggregation related to AD [26], as well as pharmacological properties in the treatment of type II diabetes [22]. Studies have shown that the potentially toxic compounds in cinnamon bark are found primarily in lipid soluble fractions but are present only in extremely low levels in water soluble cinnamon extracts [27] which are therefore considered highly safe for uptake. Here, we demonstrate the use of a natural substance based on aqueous cinnamon extract (CEppt) as an efficacious therapeutic agent that inhibits Aβ oligomer formation and ameliorates AD symptoms. In addition, we suggest the use of an efficacious platform for screening molecules as AD drugs using *in vitro* cell culture and *in vivo* AD fly and aggressive AD mouse model.

## Results

### Inhibition of toxic Aβ oligomer species and fibrils formation

**Figure 1:**
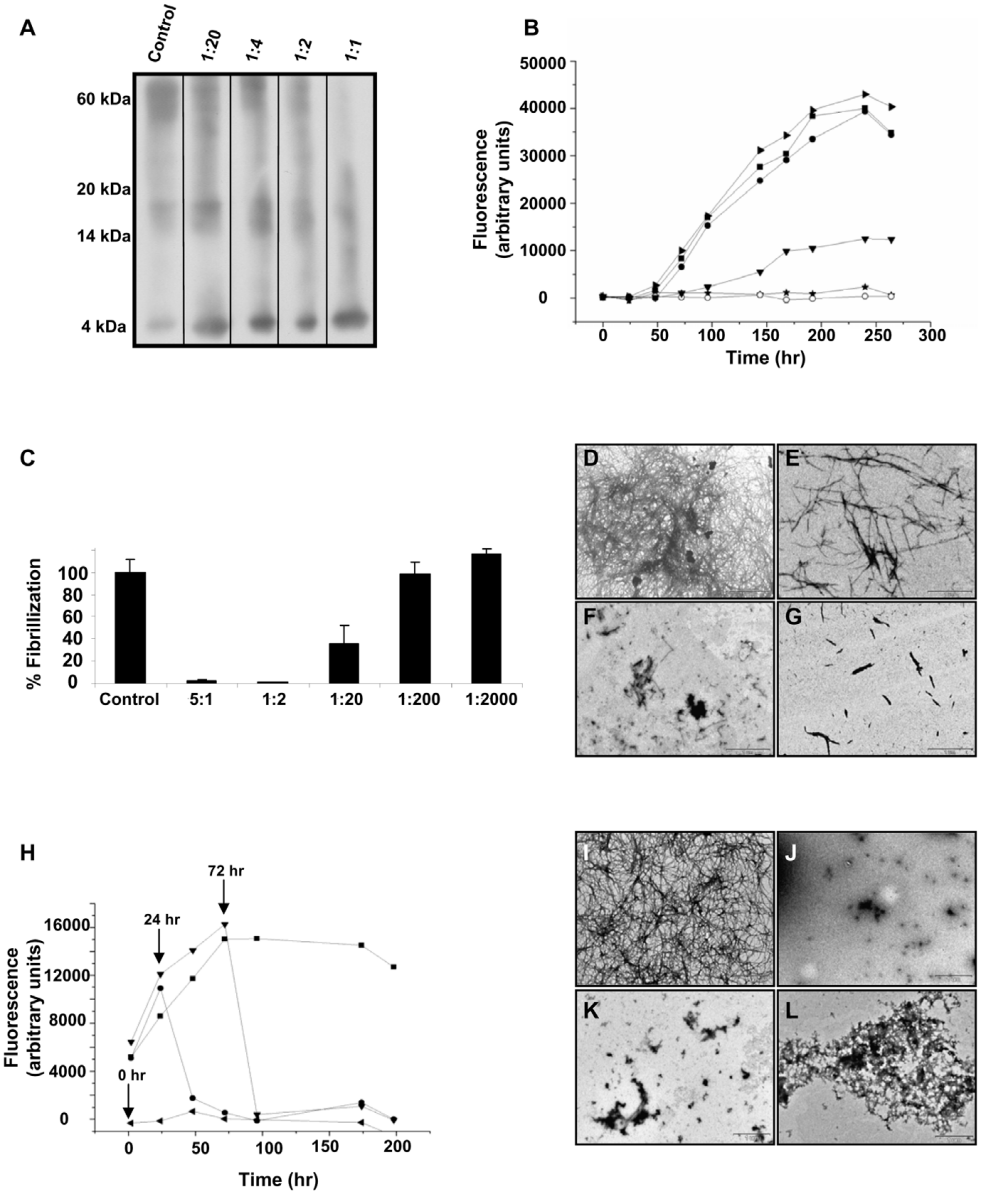
Inhibition and disaggregation of Aβ assemblies *in vitro.* (A) Determination of a dose-dependent effect of CEppt on soluble off-pathway oligomer formation. Soluble oligomers were prepared [10] with and without increasing concentration of CEppt. Concentration ratios (w/w) of CEppt: Aβ_42_ are indicated. The control is Aβ_42_ (0.6 mg/ml) alone. (B) The kinetics of Aβ_40_ (20 µg/ml) fibril formation with and without the inhibitor as assessed by the Thioflavin-T biding assay over the course of 264 hours. Concentrations are expressed as CEppt: Aβ_40_ concentration ratio (w/w). Control - Aβ_40_ only (▪); 5∶1 (*); 1∶2 (o); 1∶20 (▾); 1∶200 (•); 1∶2000 (▸). (C) Endpoint of ThT analysis measurement T = 264 hours. Concentrations are expressed as CEppt: Aβ_40_ concentration ratio (w/w), control is Aβ_40_ (20 µg/ml). (D–G) Transmission electron microscope images taken from ThT analysis after 264 hours. (D)Aβ_40_ alone, (E) CEppt:Aβ_40_ (1∶20), (F) CEppt:Aβ_40_ (1∶2), (G) CEppt:Aβ_40_ (5∶1), (H) Samples of Aβ_40_ (20 µg/ml) was incubated alone (▪) or with the addition of CEppt (100 µg/ml) at various time points of Aβ aggregation,Aβ aggregation was monitored by ThT assay. CEppt was added at 0 hour (◂); 24 hours (•) and 72 hours (▾). (I–L) Transmission electron microscope of samples taken from the disaggregation assay after 200 hr. (I) Aβ_40_ alone, (J) T = 0 hour, (K) T = 24 hours, (L) T = 72 hours.

CEppt was initially tested for its ability to inhibit Aβ_42_ oligomer formation using the protocol established by Barghorn and coworkers, which results in the formation of SDS stable off-pathway oligomers [10]. The active fraction (CEppt) was isolated as described previously [28]. Briefly, cinnamon bark was ground into powder and the active fraction was extracted from the powder into an aqueous phosphate buffer solution. CEppt fraction was then precipitated from the cinnamon extract by KCl. Aβ_42_ was incubated with increasing concentrations of CEppt, and the reaction mixtures were separated by SDS-PAGE followed by western blot analysis using a specific anti Aβ antibody (6E10) (Figure 1A). The results revealed a dose dependent inhibition of oligomer formation, where a low concentration ratio of 1∶1 (CEppt: Aβ_42_) showed total abolishment of the 56 kDa (56*) neurotoxic oligomer and an increase in the level of monomers. It seems that CEppt prevents the monomers and the early nontoxic intermediate oligomers (∼18 kDa) from further growing into the toxic 56 kDa oligomers. Furthermore, we aimed to determine the ability of CEppt to inhibit on-pathway Aβ oligomer formation. For that purpose Aβ_42_ was dissolved in DMSO followed by dilution with PBS to a final concentration of 0.4 mg/ml. The solution was incubated at 37°C and samples were taken after 1, 3 and 6 hours for evaluation in SDS gel. The results present a similar pattern of inhibition as was the outcome of the off-pathway experiment (Figure S1).

The debate regarding the comparative contribution of Aβ oligomers and fibrils to the pathogenesis of AD has not yet resolved, we therefore wanted to examine whether CEppt may inhibit the formation of Aβ fibrils as well as that of oligomers. We monitored the ability of Aβ_40_ to form amyloid fibrils in the presence or absence of CEppt for nine days using the Thioflavin-T (ThT) binding assay. While the solution of Aβ_40_ alone displayed a lag-time of about 50 hours, the lag-time of the solution containing CEppt: Aβ at concentration ratios (w/w) of 5∶1, 1∶2, 1∶20 was significantly longer (Figure 1B). Moreover, relative to the maximal fluorescence intensity reached for Aβ_40_ alone, the maximal fluorescence intensity measured for the samples containing 5∶1, 1∶2 (CEppt: Aβ showed 100% inhibition of Aβ_40_ fibrillization (Figure 1C). From the ThT experiment an IC50 of 0.7 µg/ml was calculated (Figure S2).

The inhibition of Aβ_42_ fibrillization by CEppt was also tested. Aβ_42_ peptide is the major component of senile plaque and the ratio of Aβ_42_/Aβ_40_ has been found to be increased in AD patient's brains [29]–[30]. Similar to the results using Aβ_40_, CEppt in the highest concentration was able to totally abolish fibril formation, while lower concentrations showed a tenfold decrease in CEppt's ability to inhibit the fibrillization of Aβ_42_ compared to Aβ_40_ (Figure S3). The slightly decreased inhibition ratio of CEppt may be a result of the high propensity of Aβ_42_ to rapidly aggregate *in vitro* [31].

As an additional measure for estimating the effect of CEppt on Aβ fibril formation, samples were taken from the ThT experiment (Figure 1B) after nine days of incubation and were examined by transmission electron microscopy (TEM). While the fibrils formed by Aβ alone were abundant, large, broad ribbon-like (Figure 1D), the fibrils formed by the Aβ in the presence of 1 µg/ml CEppt (CEppt: Aβ_40_, 1∶20) were of a smaller diameter and length, and were found at lower frequency (Figure 1E). Moreover, samples containing 10 µg/ml of CEppt (CEppt: Aβ_40_, 1∶2), and 100 µg/ml (CEppt: Aβ_40_, 5∶1) were devoid of fibrils and contained only small amounts of truncated fibrils (Figure F–G). These results are highly correlated with the ThT assay results.

### Inhibition/disassembly of an ongoing process of Aβ fibrillization

Presently, AD diagnosis is based on cognitive and behavioral symptoms. As these symptoms emerge relatively late in disease progression, current therapeutic intervention begins after significant neurodegeneration has occurred, thereby limiting its efficacy. Therefore, we tested whether CEppt could inhibit an ongoing process of Aβ_40_ fibrillization. To that end, a second kinetic experiment was performed, in which 100 µg/ml of CEppt was added at three different time points during the nine day period of fibrillization (0, 24 and 72 hours). Even after 72 hours, during which the process of fibrillization has already reached a plateau (Figure 1H), the addition of CEppt completely eliminated the fibrils and only a limited number of amorphous plaques were detected (Figure 1I–L).

### CEppt inhibits the cytotoxic effect of Aβ towards cultured cell line

**Figure 2:**
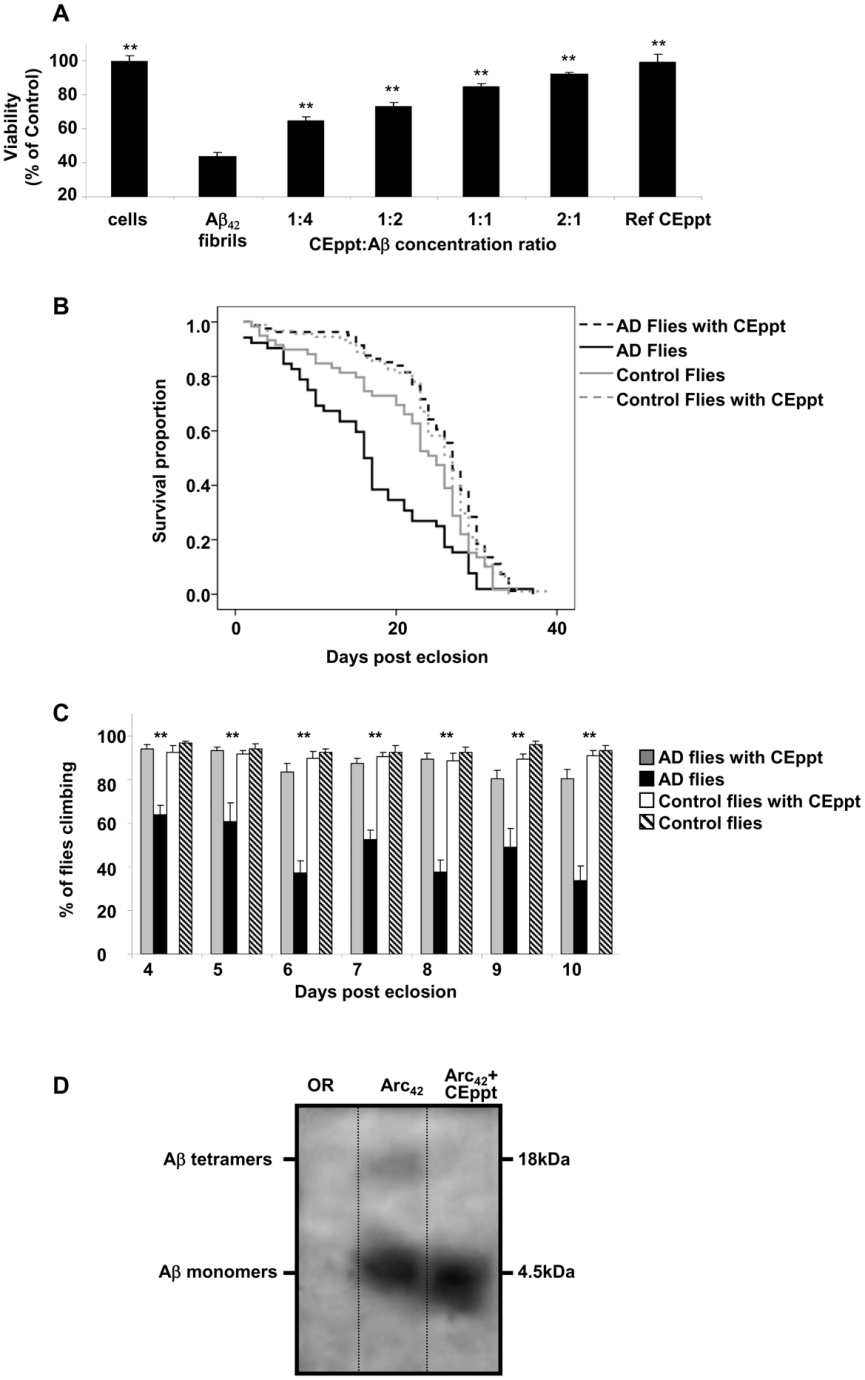
CEppt alleviates toxic effects of Aβ – cell and fly assays. (A) Samples of Aβ_42_ (20 µg/ml) with or without various concentrations of CEppt were incubated for 24 hours with PC12 cells culture. To exclude any toxic effect of CEppt, CEppt alone (9 µg/ml) was pre-incubated for 24 hours with PC12 cells (ref CEppt). Cells viability was determined using MTT viability assay. ** Pv<0.005. (B) The effect of CEppt on longevity of Aβ_42_-expressing flies. The life span of four classes of flies was evaluated. AD flies grown on regular medium (black line), AD flies grown on medium containing CEppt (black dashed line), Control Flies (carrying the Aβ_42_ transgene but not expressing it) grown on regular medium (grey line), Control Flies (carrying the Aβ_42_ transgene but not expressing it) grown on medium containing CEppt (grey dashed line). (C) The effect of CEppt on the climbing behavior of Aβ_42_-expressing flies. Four classes, each containing six-nine vials with 10 flies in each: AD flies grown on regular medium (black),AD flies grown on medium containing CEppt (grey), Control flies (carrying the Aβ_42_ transgene but not expressing it) grown on either regular medium (striped) or on medium containing CEppt (white), were analyzed using the climbing assay. Results show the percent of flies climbing to the top of the vial after 18 seconds, during the course of 10 days. Pv<0.0001. (D) Head extract from 4 days old flies. OR-control flies (left), AβArc_42_-expressing flies unfed (middle) fed with 0.75 mg/mL CEppt (right) (n = 40 in each group). Accumulation of Aβ tetramers is evident only in AβArc_42_ flies which were not fed with CEppt.

We next examined whether CEppt could inhibit Aβ-mediated cytotoxicity on the rat neuronal PC12 cell line. A control experiment showed that on its own, CEppt is not toxic to the cultured cells up to a concentration of 1 mg/ml (Figure S4 A, ref CEppt). In contrast, Aβ_42_ fibrils taken at the end of the ThT experiment (Figure S3) were highly toxic to these cells (Figure 2A). Remarkably, when incubated with Aβ_42_ fibrils, CEppt displayed a dose dependent inhibition of the cytotoxic effect of Aβ_42_ fibrils, with cell viability restored to 100% at CEppt: Aβ_40_ concentration ratio of 2∶1 (Figure 2A). Similar results were obtained when Aβ_40_ fibrils (Figure S4 A) and Aβ_42_ oligomers (Figure S4 B) were used, where very low CEppt's concentration exhibit total viability restoration of the cells.

### The effect of CEppt in an *in vivo* transgenic fly system

While CEppt exhibited dramatic abilities in the *in vitro* experiments, we wished to examine the effect of CEppt *in vivo,* by using a *Drosophila melanogaster* model. This model of transgenic flies express the human Aβ_42_ protein in their nervous system, via the Gal4-UAS system [32]. Crossing male flies carrying the pan-neuronal elav-Gal4 driver (on their X chromosome) to females carrying the UAS-regulated Aβ_42_ transgene results in AD flies expressing Aβ_42_ in their nervous system. Characteristic symptoms of these flies include impaired locomotion and memory, which decline with age, as well as markedly reduced longevity [32]. We fed flies either on regular Drosophila medium or medium supplemented with 0.75 mg/ml CEppt from the beginning of their larval stage through adulthood, and each class of adult offspring was monitored daily for survival and locomotion (climbing) ability [16], [32]–[33]. As reported, flies expressing Aβ_42_ showed a markedly reduced life span (16 days) compared to the 25 days of control flies (offspring that carried the Aβ_42_ transgene but did not express it because they lacked the Gal4 driver) (Figure 2B). The CEppt diet rectified the reduced longevity of the AD flies to a level that was not statistically different from that of the control flies (Pv = 0.0005) (Figure 2B). In contrast, CEppt had no significant effect on the life span of the control flies. CEppt also ameliorated the locomotive defects of the AD flies. While the control flies showed above 90% climbing ability at days 4–10, AD flies struggled to climb up the test tube, showing only 30%–60% climbing ability (Figure 2C). In contrast, AD flies fed with CEppt displayed climbing ability almost identical to the control flies (Figure 2C), while no effect of CEppt was observed on locomotion of the control flies at all days tested. Two- tail ANOVA statistics showed Pv = 0.0001 for all four classes. These results show a specific and marked phenotypic recovery of the AD flies fed with CEppt diet.

To Further estimate the therapeutic ability of CEppt on AD flies, Aβ was extracted from fly brains over expressing the Arctic (Arc) (E22G) mutant form of Aβ, which is associated with enhanced Aβ protofibrils formation and early-onset familial AD [34]. These flies exhibited reduced life span and deficient locomotion as described above for Aβ_42_-expressing flies [16], [32]. Low molecular species of Aβ were detected in the soluble fraction of extracts from AβArc_42_-expressing flies following immunoprecipitation with 6E10 followed by western blot. The results revealed that monomers of Aβ were not detectable in the wild type (Oregon-R) control flies (non transgenic flies that have the same genetic background as our Alzheimer's flies). In contrast, in the head extracts of CEppt-treated flies, Aβ monomers were detected and their level was increased by 20% in comparison to the non-treated AβArc_42_-expressing flies (Figure 2D). Moreover, Aβ tetramers that were present in non-treated AβArc_42_ flies [16], [32] were absent from extracts of flies fed with CEppt (Figure 2D).

### The effect of CEppt in an *in vivo* transgenic aggressive mice model

**Figure 3:**
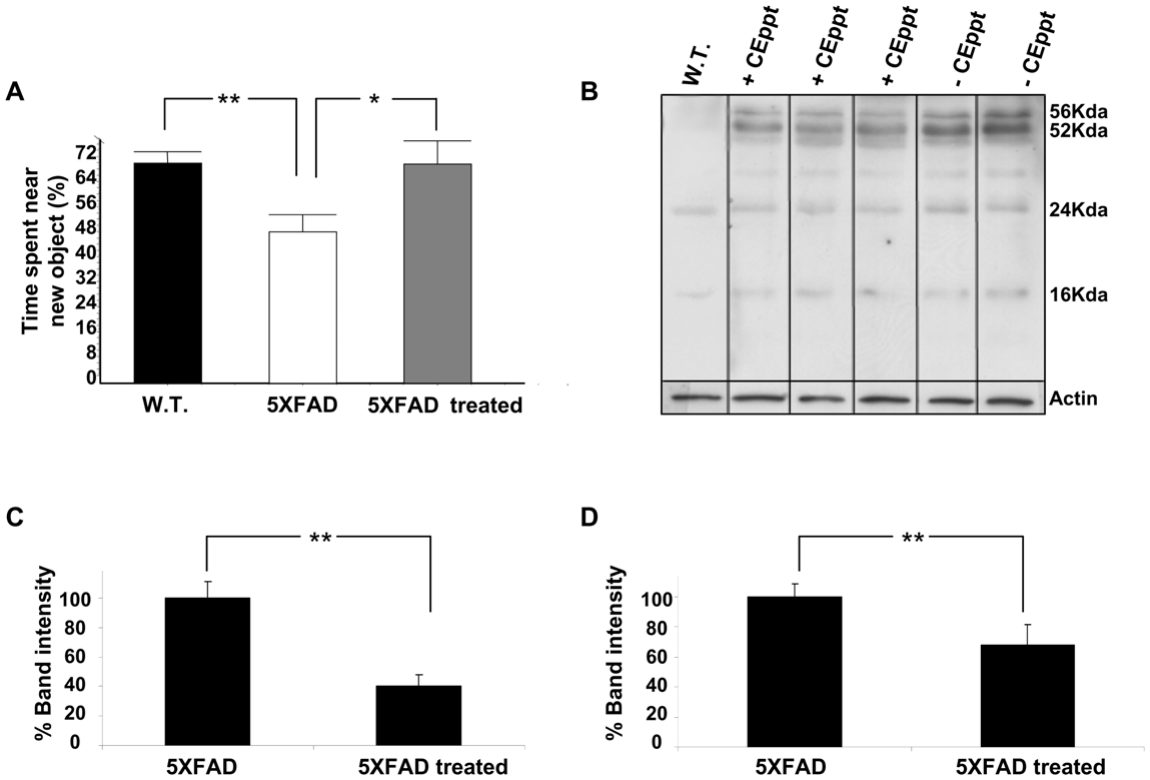
The effect of CEppt on cognitive performances and Aβ oligomer formation in 5XFAD Alzheimer's disease model mice. (A) Object recognition test of transgenic AD model mice (5XFAD), non-transgenic littermate (W.T.), and 5XFAD treated mice. Placebo 5XFAD mice were given drinking water (n = 8); W.T mice were given drinking water (n = 8); treated 5XFAD mice were given water with CEppt (100 µg/ml) (n = 7). One-way ANOVA tests show Pv = 0.0413. (B) Soluble fraction of mouse brain homogenates were analyzed by western blot probed with Aβ antibody 6E10. (C) Western blot band signals for 56 kDa in (B) were quantified using densitometry and displayed as Percent of 56 kDa toxic oligomer band intensity. (D) Insoluble fraction of mouse brain homogenates were analyzed by western blot probed with Aβ antibody 6E10, quantified using densitometry, and displayed as relative intensities of total Aβ signals.

We further examined the *in vivo* effect of CEppt on an aggressive AD mouse model [35]. The commonly used transgenic AD mice harbor three familial AD mutations [36], and have served as useful model for studying Aβ deposition, amyloidogenesis and the cognitive impairment associated with AD neuropathology. Yet, the majority of AD mice fail to develop the characteristic paired helical filaments that comprise the neurofibrillary tangles of AD and, importantly, do not show the characteristic overt neuronal loss in brain regions most affected in AD patients. Therefore, in this work we used an aggressive AD mouse model that displays the majority of AD symptoms. These transgenic mice co-express a total of five familial AD mutations (“5XFAD”) and display early (2 months) plaque formation, impaired cognition (4 months), and neuronal cell death (9 months) [35]. Mice were continuously administered with CEppt in the drinking water (100 µg/ml) from the age of two month for 120 days, and then mice were analyzed for changes in cognitive abilities, presence of oligomers and plaque loads**.** A new object recognition test [37], performed at the age of 180 days, revealed a significant improvement of memory in the CEppt-treated 5XFAD group compared to the untreated group (Pv = 0.034) (Figure 3A). Moreover, the cognitive performance of 5XFAD mice treated with CEppt was almost identical to non-transgenic mice having the same genetic background. This result does not reflect an effect on motor functions since there was no statistical difference in the ability of the 5XFAD treated and untreated mice to perform the rotarod test (data not shown). These results suggest that CEppt may have cured the defective cognition of the 5XFAD mice.

**Figure 4:**
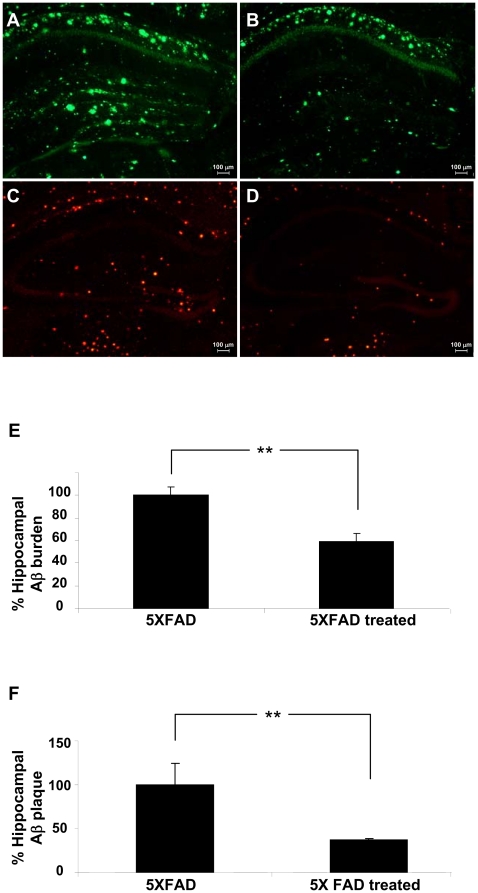
The effect of CEppt on Aβ plaques formation in 5XFAD Alzheimer's disease model mice. (A) Histological images of frontal cortex sections stained with Aβ antibody 6E10 from vehicle-treated 5XFAD mice, and (B) CEppt-treated 5XFAD mice. (C) Histological images of frontal cortex sections stained with Congo red from vehicle-treated 5XFAD mice, and (D) CEppt-treated 5XFAD mice. Scale bar = 100 µm. (E) Quantification of the % plaque load area of hippocampal sections from 5XFAD mice with or without CEppt treatment determined by 6E10 staining. (F) Quantification of the % plaque load area of hippocampal sections from 5XFAD mice with or without CEppt treatment determined by Congo red staining.

Following the behavioral assays, the animals were sacrificed and soluble and insoluble Aβ fractions were extracted from hemi-brains. Treatment with CEppt led to a remarkable reduction of 60% in the level of the 56 kDa toxic Aβ oligomer (Pv = 0.0011) (Figure 3B–C) compared to the untreated 5XFAD mice. This is the first demonstration for the ability of a natural compound to reduce the formation of the toxic 56 kDa species in an intact animal. In addition, significant reduction was observed in the number and mean size of Aβ plaque cores in the corresponding 5XFAD hemi-brains. Frontal sections of the hippocampus treated vs. untreated mice were stained with the Aβ-specific 6E10 antibody and revealed a reduction of 42% in plaque load following treatment with CEppt (Pv = 0.0043) (Figure 4A–B, E). Furthermore, when stained with Congo red, a specific amyloid-binding dye, a 63% reduction in amyloid plaques was observed (Pv = 0.034) (Figure 4C–D, F). Aβ reduction of about 35% was calculated from the total insoluble Aβ fraction as determined by western blot analysis (Pv = 0.021) (Figure 3D). Taken together, these results suggest that treatment with CEppt reduced Aβ deposition and improved cognitive function in 5XFAD mice.

## Discussion

AD is the most common cause of late-life dementia and a leading cause of death in the western world. While the etiology of AD is not fully understood, compelling evidences has accumulated over the past few years for a correlation between soluble Aβ and the extent of synaptic loss and cognitive impairment [1], [4], [6]–[8]. Thus, it appears that the most promising strategy for developing therapeutic agents to treat AD and other amyloid-associated diseases is by targeting the early molecular recognition and self-assembly processes [14], [16].

Here, we have identified a novel herbal substance, based on aqueous cinnamon extract (CEppt) that can serve as an efficacious inhibitor of both the oligomerization and fibrillization of Aβ *in vitro* and *in vivo*. In contrast to the raw cinnamon bark, which is considered to have hepatotoxic effects [27], our method of preparation results in a highly potent non-toxic compound which is easily manufactured at low cost.

Pervious studies have already demonstrated the powerful usage of herbal extract in arresting amyloid fibrils. Examples for extensively studied naturally occurring compounds are the (-)-epigallocatechin-3-gallate (EGCG) from green tea and Curcumin which is derived from the natural turmeric. Both EGCG and Curcumin have been reported in several studies as a potential neuroprotective strategy for neurodegenerative disorder such as AD and Parkinson disease (PD) [38]–[39]. Yet, to the best of our knowledge, no study has yet demonstrated the ability of a natural substance to reduce the level of toxic 56 kDa oligomers, which are thought to be a major neurotoxic species of Aβ [7], [10].

In order to determine the pattern of inhibition and to understand which phase of the amyloidogenic process is inhibited by CEppt, we performed various analyses and followed the kinetics of amyloid fibrils formation and their morphology. CEppt changed the shape of the nucleation curve, causing a longer lag- phase and a considerably lower amount of amyloid fibril formation (Figure 1B–G, Figure S3). In addition, we demonstrated that CEppt stabilizes the non-toxic early off- pathway oligomers and prevents their further assembly into the toxic 56 kDa oligomer species (Figure 1A), as well as inhibiting the early formation of on- pathway oligomer assemblies (Figure S1). Furthermore, we found that CEppt can arrest an ongoing process of Aβ fibrillization and to disassemble preformed fibrils, a characteristic which is very important when dealing with neurodegenerative disorders whose symptoms emerge relatively late in the disease progression when fibrils have already begun to form. Importantly, the inhibitory effects of CEppt on Aβ assembly *in vitro* correlate well with its effects *in vivo*, where CEppt completely alleviated Aβ-engendered symptoms in transgenic fly and mice models of AD.

In contrast to the majority of AD transgenic mice models, in which formation of amyloid plaques takes about 6–12 months [36], we used an aggressive 5XFAD mice [35]. These model mice display large amyloid deposition burden at a very early age (2 month), and exhibit intraneuronal Aβ_42_, neurodegeneration and neuronal loss [35]. These characteristics could be a disadvantage for evaluating drug candidates that are moderately efficacious and could be overlooked when tested in such an aggressive model, as opposed to the common more moderate models which may recapitulate the slower progression of AD in humans. However, the fact that CEppt caused a significant reduction of the amount of amyloid deposits and of the soluble 56 kDa toxic oligomer in the brains of the 5XFAD mice, and dramatically improved their cognitive performance suggests that CEppt may be likewise effective in the more typical AD mice models and in human patients.

Several reports have indicated the presence of polyphenol in the cinnamon extract [27], [40]. Moreover, several studies have shown that polyphenols can inhibit the aggregation of various amyloidogenic peptides [41]–[43]. We hypothesize that CEppt may interacts with the Aβ peptide at a very early stage of its self assembly through the polyphenol entity to inhibit its aggregation, thereby preventing the Aβ- mediated toxicity.

Although these results clearly show the ability of CEppt to inhibit the progress of Aβ aggregation, it is important to note that the mechanism of action remains to be elucidated. Moreover, in this work, the ability of CEppt to cross the BBB was not tested. It is possible that several active molecules in CEppt or their derivatives may cross the BBB, yet it is possible that CEppt possess a peripheral mode of therapy. Since CEppt is comprised of several molecules, characterizing the active compound and understanding the mechanism of action will require further work.

To conclude, we have identified a novel prophylactic approach for AD, utilizing a natural substance, which is currently very common in human diet. The CEppt fraction is orally bioavailable and very safe *in vitro* and *in vivo.* We demonstrated that a minimal concentration of CEppt is capable of inhibiting the formation of both Aβ toxic oligomer species and amyloid fibrils in both the test tube and in the intact animal, and can correct the cognitive impairment in AD animal models. The agreement between the results obtained *in vitro*, in cultured cells, in flies and in mice suggests that together they can be used as an efficient platform for screening molecules as drugs for the treatment of AD. Moreover, we believe that the CEppt, which has a unique herbal supplement profile of actions with multiple components, would have an advantage over crystal-purified drugs containing only a single component, and could be developed as novel precluding, easy to administer, prophylactic treatment of AD pathogenesis.

## Materials and Methods

### Ethics Statement

The mice were housed and maintained in the animal facility of Tel Aviv University, and all experiments were in compliance with protocols approved (Permit Number is: L-10-027) by the TAU animal care committee.

### Cinnamon extract

Cinnamon bark was ground into powder using a coffee grinder. The active material was extracted from the powder into an aqueous phosphate buffer solution 0.02 M, pH 7 overnight and centrifuged. The supernatant was kept at 4°C until use. CEppt fraction was then precipitated from the cinnamon extract (CE) by KCL 0.3 M and dissolved in 0.02 M phosphate buffer, pH 7, or in water, as needed.

### Aβ solution

Synthetic lyophilized Aβ_40_ or Aβ_42_ (Bachem, Bubendorf, Switzerland) was dissolved in dimethylsulfoxide (DMSO) to a concentration of 0.4 mg/ml and sonicated in ice water for 20 sec to avoid pre-aggregation. Aβ solutions were prepared by immediate dilution with 10 mM phosphate-buffered saline (100 mM NaCl, 0.5 mM EDTA, pH 7.4) to a concentration of 40 µg/ml and further diluted with CEppt solution to final concentration of 20 µg/ml (containing 5% (v/v) DMSO).

### Thioflavin T binding fluorescence

CEppt was dissolved in ddH2O to a concentration of 10 mg/ml and then diluted with 10 mM PBS buffer, (100 mM NaCl, 0.5 mM EDTA, pH 7.4) to a final concentration of (0.01 µg/ml–1 mg/ml). 40 µg/ml Aβ_40_ or Aβ_42_ solution was immediately mixed with the cinnamon solutions to final concentration of 20 µg/ml Aβ and various cinnamon concentrations (0.01 µg/ml–100 µg/ml). The samples were incubated at 37°C and the fibrillogenesis rate was monitored using ThT fluorescence analysis. The respective excitation and emission wavelengths were 450 nm, 2.5 nm slit, and at 480 nm, 5 nm slit. A 10-fold diluted sample was taken and mixed with 900 µl ThT 0.4 µM. The fluorescence of ThT was measured using a Jobin Yvon Horiba Fluoromax 3 fluorimeter. Each experiment was repeated in quadruplicate.

### Disaggregation assay

Synthetic lyophilized Aβ_40_ (Bachem, Bubendorf, Switzerland) was dissolved in dimethylsulfoxide (DMSO) to a concentration of 0.4 mg/ml and sonicated in ice water for 20 sec to avoid pre-aggregation. Aβ solution was prepared by immediate dilution with 10 mM phosphate-buffered saline (100 mM NaCl, 0.5 mM EDTA, pH 7.4) to a final concentration of 40 µg/ml (containing 10% DMSO). CEppt was dissolved in ddH2O to a concentration of 200 µg/ml and then diluted with the Aβ solution to a final concentration of 100 µg/ml, same amount of ddH2O was added to the W.T sample as a reference. The CEppt was added at various time points- 0 hour, 24 hours and 72 hours. Samples were monitored using ThT assay as describe above.

### Transmission electron microscopy

10 µl from different ThT fluorescence samples were placed on 400 mesh copper grids covered by carbon-stabilized Formvar film (SPI Supplies, West Chester, PA). After 1.5 minutes, excess fluid was removed, and the grids were negatively stained with 10 µl of 2% uranyl acetate solution for 1.5 min. Finally, excess fluid was removed and the samples were viewed in a JEOL 1200EX electron microscope operating at 80 kV.

### Determination of off-pathway soluble oligomer formation

Aβ_42_ intermediates and toxic oligomers (56 kDa) were produced according to Barghorn *et al, 2005* [10]. To avoid pre-aggregation, synthetic lyophilized Aβ_42_ was pretreated with Hexafluoro-2-propanol (HFIP). Aβ_42_ was dissolved in 100% HFIP, sonicated in ice water for 20 seconds and incubated for 2 hours at 37°C under shaking at 100 RPM. The inhibitor CEppt was dissolved in DMSO to a concentration of 40 mg/ml, sonicated for 1 min and then diluted in DMSO to several concentrations (1 mg/ml–20 mg/ml). After evaporation in a speedVac, Aβ_42_ was resuspended in DMSO (with or without the CEppt in different concentrations) to 20 mg/ml and diluted with PBS (20 mM NaH_2_PO_4_, 140 mM NaCl, pH 7.4) to a final concentration of 2 mg/ml and 1/10 volume 2% SDS (final concentration of 0.2%). The Aβ toxic oligomers were generated by a further dilution with H_2_O to final concentration of 0.6 mg/ml and incubated for another 18 hours or more. Aβ aggregation products were then separated using a 15% Tris-Tricine gel followed by western blot analysis by a specific anti Aβ antibody (6E10) (SIGNET).

### Determination of on-pathway soluble oligomer formation

To avoid pre-aggregation, synthetic lyophilized Aβ_42_ was dissolved in 100% HFIP, sonicated in ice water for 20 seconds and incubated for 2 hours at 37°C under shaking at 100 RPM. After evaporation in a speedVac, Aβ_42_ was resuspended in DMSO to a concentration of 9 mg/ml and then diluted with or without CEppt at different concentrations to a final concentration of 0.4 mg/ml. The samples were incubated for 6 hours at 37°C. Aβ aggregation products were then separated using a 15% Tris-Tricine gel followed by western blot analysis using a specific anti Aβ antibody (6E10) (SIGNET).

### Cells cytotoxicity assay

PC12 pheochromocytoma cell line was routinely grown in Dulbecco's Modified Eagle Medium (DMEM) supplemented with 8% Fetal Calf Serum, 8% horse serum, 100 U/ml penicillin, 100 U/ml streptomycin and 2 mM L-glutamine.

Sub-confluent cells were harvested by trypsinization, counted and diluted in the cell media to 2×10^4^ cells/ml, then cultured in 96 wells plate (100 µl/well) and incubated over-night at 37°C. In order to exclude the effect of the serum, the wells were washed once with serum free-DMEM. Then, into each well added 100 µl of DMEM with Aβ_40_ or Aβ_42_ fibrils 20 µg/ml previously incubated with or without CEppt (as describes above) or Aβ_42_ oligomers that were incubated previously with or without CEppt. The Aβ_42 _oligomers were first precipitated with acetone to exclude toxicity of the SDS and HFIP from the oligomer preparation protocol of Barghorn *et al,* [10]. Each treatment was performed in four repeats. After 24 hr incubation at 37°C, cell viability was evaluated using thiazolyl-blue-tetrazolium-bromide (MTT) assay. Briefly, 20 µl of 5 mg/ml MTT dissolved in PBS were added into each well. After 4 hr incubation at 37°C, 100 µl of extraction buffer (20% SDS dissolved in 50% dimethylformamide and 50% DDW solution, pH 4.7) were added into each well and the plates were incubated again overnight at 37°C. Finally, color intensity was measured using ELISA Reader at 570 nm.

Cells viability (%)  =  O.D. (570 nm) in the presence of Aβ with or without inhibitor*100, and O.D. (570 nm) when only inhibitor was added.

### Fly keeping

Flies were reared on standard corneal-molasses medium and were kept at 25°C. As Drosophila females can store sperm cells in their bodies, crosses were conducted using virgin females collected no longer than 8 hours after eclosion at 25°C or 18 hours after eclosion at 18°C. Adult offspring (F1) from the crosses were collected up to 9 days after the beginning of their eclosion at 25°C in order to avoid offspring from the next generation (F2).

### Fly crossing

Male flies carrying the driver elav^c155^-Gal4 (on their X chromosome) were crossed with females carrying the Aβ_42_ transgene (located on an autosome) under the UAS promoter in a homozygous condition. This resulted in first generation (F1) female offspring expressing Aβ_42_ in their nervous system. They served as our Alzheimer's Drosophila model. Male F1 offspring, which carried the Aβ_42_ transgene but did not express it (because they lacked the Gal4 driver) served as a control.

### Special fly feeding

CEppt dissolved in ddH2O was added to standard corneal-molasses medium about 10 minutes after cooking (0.75 mg/mL). The compound was mixed thoroughly into the medium and the mixture was aliquoted into rearing vials. The vials were kept at 4°C until use. Crosses were performed either on regular Drosophila medium (control) or on medium supplemented with CEppt. Animals fed on the appropriate medium from the beginning of the larval stage onwards.

### Longetivity assay

Flies expressing one copy of Aβ_42_ reared at 29°C on medium with or without CEppt were separated to four classes: 1. Females expressing Aβ_42_, on regular medium. 2. Females expressing Aβ_42_, on medium supplemented with CEppt. 3. Male controls (lacking the Gal4 driver), on regular medium. 4. Male controls (lacking the Gal4 driver), on medium supplemented with CEppt. For each class, six plastic vials each with 10 flies were collected and fresh food was given every three days (whether with or without CEppt). The number of viable Aβ-expressing and control flies treated with and without CEppt was recorded daily post eclosion. Differences in survival rates were analyzed using the SPSS 11 Kaplan-Meir software package.

### Locomotive (climbing) assay

Test tubes of each of the four classes mentioned above, each containing 10 flies were tapped gently on the table and were let stand for 18 seconds. The percent of flies which climbed to the top of the test tube was then calculated over time. Each class had six independent test tube repeats. Statistical analysis was done using StatSoft Statistica 7, parametric ANOVA testing.

### Immuno-precipitation and western-blot of fly head extracts

Forty freshly decapitated heads from 4 day old OR-control, AβArc_-42_ flies treated and non-treated with CEppt (as described before) were collected and homogenized in 30 µl PBS/protease inhibitor/1% SDS [16], [32]. Homogenates were then centrifuged at 13,000 rpm for 25 seconds and the supernatant was further immuno-precipitated with specific 6E10 anti-Aβ antibody (1∶10) over night at 4°C. Samples were boiled for 6 minutes, and were then western blotted and membranes were boiled in PBS for 10 minutes before antibodies were introduced. Total protein levels of the samples were quantified using Bradford analysis and a total of 100 µg protein were loaded in each lane. Since samples were loaded after IP with specific 6E10 anti-Aβ antibody, no marker protein levels could be measured.

### Object recognition test

After 4 months of treatment with 100 µg/ml CEppt solution or water, the mice were tested using a novel object recognition test (ORT). Object recognition is distinguished by more time spent interacting with the novel object [37]. Memory was operationally defined by the discrimination index for the novel object (DI) as the proportion of time the mice spent investigating the novel object and the familiar one.

### Immunohistology

6-month-old 5XFAD mice were sacrificed (transcardially punctured and saline-perfused) and their brains rapidly excised and frozen. The brains (left hemisphere) were cut in 14 µm coronal brain sections, using cryostat at −20°C, and used for histological examination. The slices were stained at Bregma −1.58 mm with Congo-Red staining [Sigma-C6767] and Anti Aβ (SIG-39320500 R&D) and visualized by fluorescence microscopy for quantification of the amount of vascular amyloid depositions. Quantification of hippocampus Aβ burden was done in a blinded fashion way using Imaging Research software from the NIH in an unbiased stereological approach.

### Soluble and insoluble Aβ brain quantification

Right hemisphere of each mouse was homogenized in 1 ml protease inhibitor dissolved in PBS. Homogenates were then centrifuged at 113,000 g for 40 minutes at 4°C, and two fractions were obtained: soluble and insoluble. The insoluble fraction was dissolved in 1 ml 5 M Guanidine Hydrochloride, 50 mM Tris, pH 8 and was incubated over night in room temperature with gentle agitation. Soluble and insoluble fractions were then quantified by Bradford reagent. An aliquot of each sample was resolved by SDS-PAGE and transferred using standard semi-dry conditions. Proteins were resolved by 12% SDS-PAGE and transferred onto a 0.45 µm nitrocellulose membrane at 100 V for 30 min. (Bio-Rad) using a Semi-Dry Electroblotter (Bio-Rad). After transfer, the membrane was blocked overnight in 5% (wt/vol) nonfat dry milk in TBS-T (20 mM Tris, pH 7.4, 150 mM NaCl, 0.1% (vol/vol) Tween 20) and washed 3 times for 10 min each in TBS-T before and after adding the antibody. All antibodies were diluted in 5% (wt/vol) nonfat dry milk in TBS-T. The membrane was incubated with 6E10 antibody (1∶1000) (SIGNET) for 2 h at room temperature. After wash with TBS-T, the blots were incubated with Anti-Mouse HRP secondary antibodie (1∶10,000) (Jackson immunoresearch laboratories) for 1 h at room temperature and developed with enhanced chemiluminescence (ECL) reagents (Pierce). The protein band was visualized using X-ray film. Gels were analyzed using densitometry and bands were normalized to Actin.

### Statistical Analysis

Data comparisons were performed using the Student's t test when two groups were compared or one-way ANOVA analysis of variance when three or more groups were analyzed. A *P* value of <0.05 was considered significant. *, Pv<0.05, **, Pv<0.005.

## Supporting Information

Figure S1 **Determination of a dose-dependent effect of CEppt on on-pathway Aβ_42_ soluble oligomer formation.** Soluble oligomers were prepared with or without increasing concentration of CEppt. Concentration ratios (w/w) of Aβ_42_: CEppt are indicated. The control is Aβ_42_ 0.4 mg/ml alone. Samples were loaded on SDS gel after 1, 3 and 6 hours followed by western blot with 6E10. By 1 hour concentration ratios of 1∶2 and 1∶1 (Aβ_42_: CEppt) could dramatically inhibit the higher MW oligomers (∼60–80 kDa) while increasing the level of monomers and low MW oligomers (4–10 kDa). At lower concentration ratios of 2∶1 and 4∶1 (Aβ_42_: CEppt) the formation of higher MW oligomers was still inhibited while the level of the monomers and low MW oligomers was decreased, yet an increment of intermediate oligomers (15–20 kDa) was observed. By 3 hours the monomers were less affected but there still was a very efficacious inhibition of the higher MW oligomers while the intermediate oligomers were increased at ratios of 1∶2 and 1∶1 Aβ_42_: CEppt. By 6 hours the main species that were inhibited were high MW and protofibrils.
(PDF)

Figure S2 **Concentration-dependent inhibition of Aβ_40_ fibrillogenesis.** CEppt was added at different concentrations to a fixed amount of 20 µg/ml Aβ_40_. After incubation for 264 hours at 37°C, ThT fluorescence was monitored at an emission wavelength of 480 nm (excitation at 450 nm).
(PDF)

Figure S3 **Inhibition of Aβ_42_ assemblies** ***in vitro.*** (A) The kinetics of Aβ_42_ (20 µg/ml) fibril formation in the absence or presence of CEppt as assessed by the Thioflavin-T binding assay over the course of 312 hours. Concentrations are expressed as CEppt:Aβ_42_ concentration ratio (w/w). Control - Aβ_42_ only (▪); 5∶1 (*); 1∶2 (o); 1∶20 (▴); 1∶200 (-); 1∶2000 (•). (B) Endpoint of ThT analysis measurement T = 312 hours. Concentrations are expressed as CEppt: Aβ_42_ concentration ratio (w/w), control is Aβ_42_ (20 µg/ml).
(PDF)

Figure S4 **CEppt alleviates toxic effects of Aβ oligomers and fibrils in cell assay.** (A) Samples of Aβ_40_ (20 µg/ml) with or without various concentrations of CEppt were incubated for 24 hours with PC12 cells culture. To exclude any toxic effect of CEppt, CEppt alone (1 mg/ml) was pre-incubated for 24 hours with PC12 cells (ref CEppt). Cells viability was determined using MTT viability assay. (B) Samples of Aβ_42_ oligomers (0.6 mg/ml) with or without various concentrations of CEppt were incubated for 24 hours with PC12 cells culture. To exclude any toxic effect of CEppt, CEppt alone at the highest concentration was pre-incubated for 24 hours with PC12 cells (ref CEppt). Cells viability was determined using MTT viability assay. * Pv< 0.05, ** Pv<0.005.
(PDF)

## References

[1] Querfurth HW, LaFerla FM (2010). Alzheimer's disease.. N Engl J Med.

[2] Van Leuven F (2000). Single and multiple transgenic mice as models for Alzheimer's disease.. Prog Neurobiol.

[3] Gazit E (2004). The role of prefibrillar assemblies in the pathogenesis of amyloid diseases.. Drugs Fut.

[4] De Felice FG, Vieira MN, Saraiva LM, Figueroa-Villar JD, Garcia-Abre J (2004). Targeting the neurotoxic species in Alzheimer's disease: inhibitors of A beta oligomerization.. FASEB J.

[5] Kayed R, Head E, Thompson JI, MeIntire TM, Milton SC (2003). Common structure of soluble amyloid oligomers implies common mechanism of pathogenesis Science.

[6] Cleary JP, Walsh DM, Hofmeister JJ, Shankar GM, Kuskowski MA (2005). Natural oligomers of the amyloid-β protein specifically disrupt cognitive function.. Nat Neuroscience.

[7] Lesne S, Koh MT, Kotilinek L, Kayed R, Glabe CG (2006). A specific amyloid –β protein assembly in the brain impairs memory.. Nature.

[8] Walsh DM, Selkoe DJ (2007). A beta oligomers - a decade of discovery.. J Neurochem.

[9] Hardy J, Selkoe DJ (2002). The amyloid hypothesis of Alzheimer's disease: progress and problems on the road to therapeutics.. Science.

[10] Barghorn S, Nimmrich V, Striebinger A, Krantz C, Keller P (2005). Globular amyloid beta-peptide oligomer - a homogenous and stable neuropathological protein in Alzheimer's disease.. J Neurochem.

[11] Prins ND, Visser PJ, Scheltens P (2010). Can novel therapeutics halt the amyloid cascade?. Alzheimers Res Ther.

[12] Abramov E, Dolev I, Fogel H, Ciccotosto GD, Ruff E (2009). Amyloid-beta as a positive endogenous regulator of release probability at hippocampal synapses.. Nat Neurosci.

[13] Kim W, Kim Y, Min J, Kim DJ, Chang Y (2006). A high-throughput screen for compounds that inhibit aggregation of the Alzheimer's peptide.. ACS Chem Biol.

[14] Frydman-Marom A, Rechter M, Shefler I, Bram Y, Shalev DE (2009). Cognitive-performance recovery of Alzheimer's disease model mice by modulation of early soluble amyloidal assemblies.. Angew Chem Int Ed Engl.

[15] Frenkel D, Puckett L, Petrovic S, Xia W, Chen G (2008). A nasal proteosome adjuvant activates microglia and prevents amyloid deposition.. Ann Neurol.

[16] Scherzer-Attali R, Pellarin R, Convertino M, Frydman-Marom A, Egoz-Matia N (2010). Complete phenotypic recovery of an Alzheimer's disease model by a quinone-tryptophan hybrid aggregation inhibitor.. PLoS One.

[17] Paiva SAR, Russell RM (1999). {beta}-Carotene and Other Carotenoids as Antioxidants.. J Am Coll Nutr.

[18] Mee Young H, Navindra PS, Yanjun Z, David H (2008). Anticancer effects of Chinese red yeast rice versus monacolin K alone on colon cancer cells.. J Nutr biochem.

[19] Chauhan NB, Sandoval J (2007). Amelioration of early cognitive deficits by aged garlic extract in Alzheimer's transgenic mice.. Phytother Res.

[20] Ono K, Condron MM, Ho L, Wang J, Zhao W (2008). Effects of grape seed-derived polyphenols on amyloid beta-protein self-assembly and cytotoxicity.. J Biol Chem.

[21] Kim DS, Kim JY, Han YS (2007). Alzheimer's disease drug discovery from herbs: neuroprotectivity from beta-amyloid (1-42) insult.. J Altern Complement Med.

[22] Dugoua JJ, Seely D, Perri D, Cooley K, Forelli T (2007). From type 2 diabetes to antioxidant activity: a systematic review of the safety and efficacy of common and cassia cinnamon bark.. Can J Physiol Pharmacol.

[23] Khan A, Safdar M, Ali Khan MM, Khattak KN, Anderson RA (2003). Cinnamon improves glucose and lipids of people with type 2 diabetes.. Diabetes Care.

[24] Brahmachari S, Jana A, Pahan K (2009). Sodium benzoate, a metabolite of cinnamon and a food additive, reduces microglial and astroglial inflammatory responses.. J Immunol.

[25] Ouattara B, Simard RE, Holley RA, Piette GJ, Begin A (1997). Antibacterial activity of selected fatty acids and essential oils against six meat spoilage organisms.. Int J Food Microbiol.

[26] Peterson DW, George RC, Scaramozzino F, LaPointe NE, Anderson RA (2009). Cinnamon extract inhibits tau aggregation associated with Alzheimer's disease in vitro.. J Alzheimers Dis.

[27] Anderson RA, Broadhurst CL, Polansky MM, Schmidt WF, Khan A (2004). Isolation and characterization of polyphenol type-A polymers from cinnamon with insulin-like biological activity.. J Agric Food Chem.

[28] Sevillia G, Kamensky M, Finger A, Ovadia M (2007). Cinnamon Extract Inhibits Avian Influenza H9N2 Both In-Vitro and In-Vivo.. Options for the control of Influenza VI Proceedings.

[29] Gravina SA, Ho L, Eckman CB, Long KE, Otvos L (1995). Amyloid beta protein (A beta) in Alzheimer's disease brain. Biochemical and immunocytochemical analysis with antibodies specific for forms ending at A beta 40 or A beta 42(43).. J Biol Chem.

[30] Roher AE, Lowenson JD, Clarke S, Woods AS, Cotter RJ (1993). beta-Amyloid-(1-42) is a major component of cerebrovascular amyloid deposits: implications for the pathology of Alzheimer disease.. Proc Natl Acad Sci U S A.

[31] Jarrett JT, Berger EP, Lansbury PT (1993). The C-terminus of the beta protein is critical in amyloidogenesis.. Ann N Y Acad Sci.

[32] Crowther DC, KIinghorn KJ, Miranda E, Page R, Curry JA (2005). Intraneuronal Aβ, non-amyloid aggregates and neurodegeneration in a drosophila model of Alzheimer's disease.. Neuroscience.

[33] Moloney A, Sattelle DB, Lomas DA, Crowther DC (2009). Alzheimer's disease: insights from Drosophila melanogaster models.. Trends Biochem Sci.

[34] Nilsberth C, Westlind-Danielsson A, Eckman CB, Condron MM, Axelman K (2001). The 'Arctic' APP mutation (E693G) causes Alzheimer's disease by enhanced Abeta protofibril formation.. Nat Neurosci.

[35] Oakley H, Cole SL, Logan S, Maus E, Shao P (2006). Intraneuronal beta-Amyloid Aggregates, Neurodegeneration, and Neuron Loss in Transgenic Mice with Five Familial Alzheimer’s Disease Mutations: Potential Factors in Amyloid Plaque Formation.. J Neurosci.

[36] Spires TL, Hyman BT (2005). Transgenic models of Alzheimer's disease: learning from animals.. NeuroRx.

[37] Bevins RA, Besheer J (2006). Object recognition in rats and mice: a one-trial non-matching-to-sample learning task to study 'recognition memory'.. Nat Protoc.

[38] Ehrnhoefer DE, Bieschke J, Boeddrich A, Herbst M, Masino L (2008). EGCG redirects amyloidogenic polypeptides into unstructured, off-pathway oligomers.. Nat Struct Mol Biol.

[39] Hamaguchi T, Ono K, Yamada M (2010). Curcumin and Alzheimer's Disease.. CNS Neurosci Ther ahead of print.

[40] Panickar KS, Polansky MM, Anderson RA (2009). Cinnamon polyphenols attenuate cell swelling and mitochondrial dysfunction following oxygen-glucose deprivation in glial cells.. Exp Neurol.

[41] Porat Y, Mazor Y, Efrat S, Gazit E (2004). Inhibition of Islet amyloid polypeptide fibril formation: a potential role for heteroaromatic interactions.. Biochemistry.

[42] Bastianetto S, Krantic S, Quirion R (2008). Polyphenols as potential inhibitors of amyloid aggregation and toxicity: possible significance to Alzheimer's disease.. Mini Rev Med Chem.

[43] Shoval H, Weiner L, Gazit E, Levy M, Pinchuk I (2008). Polyphenol-induced dissociation of various amyloid fibrils results in a methionine-independent formation of ROS.. Biochim Biophys Acta.

