# Supplementary figures and images for: Orally Administrated Cinnamon Extract Reduces β-Amyloid Oligomerization and Corrects Cognitive Impairment in Alzheimer's Disease Animal Models
Source: PLoS One. 2011 Jan 28;6(1):e16564. doi: 10.1371/journal.pone.0016564 (PMC3030596; doi:10.1371/journal.pone.0016564)

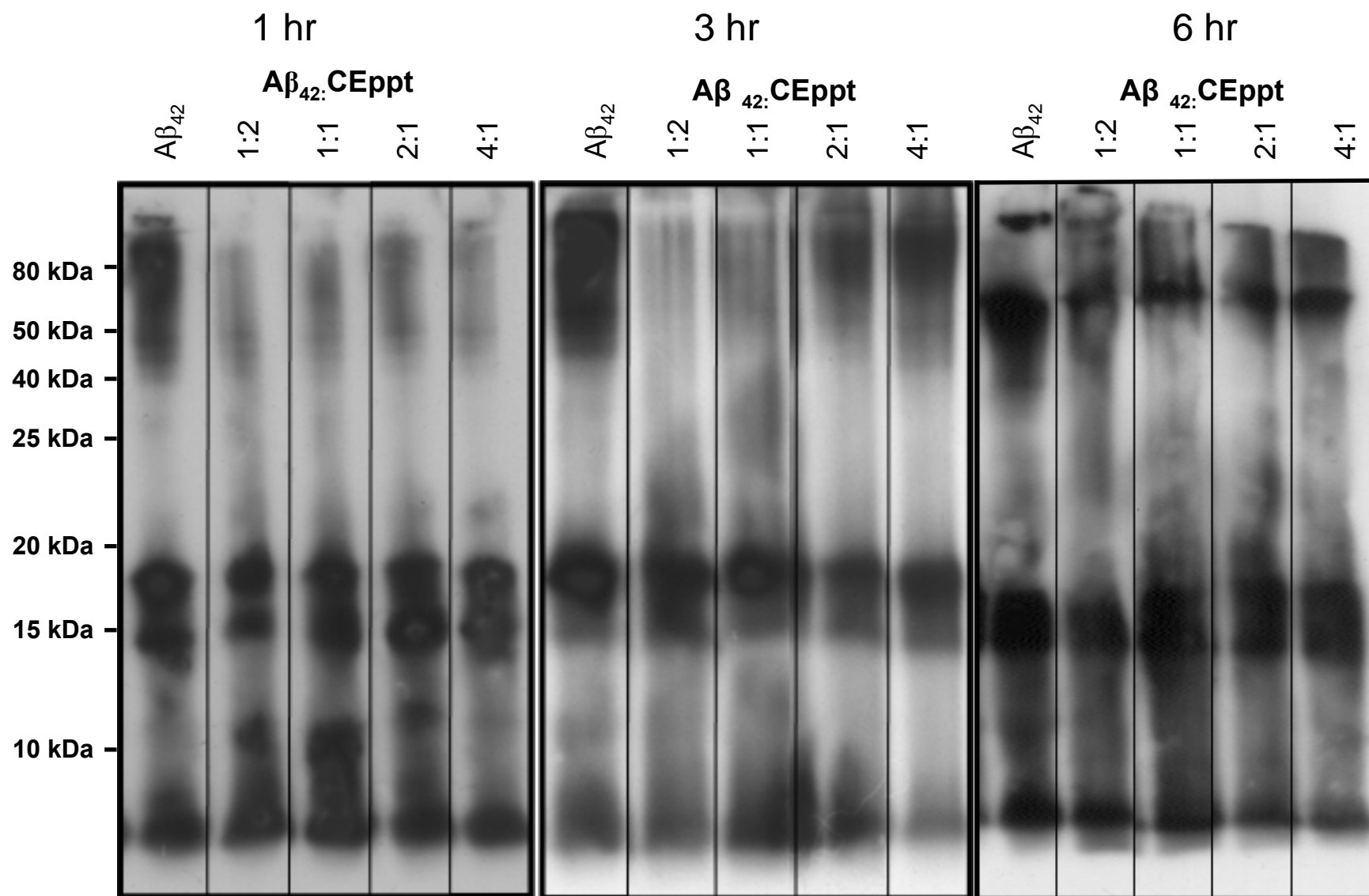

Supplement: Figure S1 — Determination of a dose-dependent effect of CEppt on on-pathway Aβ42 soluble oligomer formation. Soluble oligomers were prepared with or without increasing concentration of CEppt. Concentration ratios (w/w) of Aβ42: CEppt are indicated. The control is Aβ42 0.4 mg/ml alone. Samples were loaded on SDS gel after 1, 3 and 6 hours followed by western blot with 6E10. By 1 hour concentration ratios of 1∶2 and 1∶1 (Aβ42: CEppt) could dramatically inhibit the higher MW oligomers (∼60–80 kDa) while increasing the level of monomers and low MW oligomers (4–10 kDa). At lower concentration ratios of 2∶1 and 4∶1 (Aβ42: CEppt) the formation of higher MW oligomers was still inhibited while the level of the monomers and low MW oligomers was decreased, yet an increment of intermediate oligomers (15–20 kDa) was observed. By 3 hours the monomers were less affected but there still was a very efficacious inhibition of the higher MW oligomers while the intermediate oligomers were increased at ratios of 1∶2 and 1∶1 Aβ42: CEppt. By 6 hours the main species that were inhibited were high MW and protofibrils. (PDF) [file pone.0016564.s001.pdf]

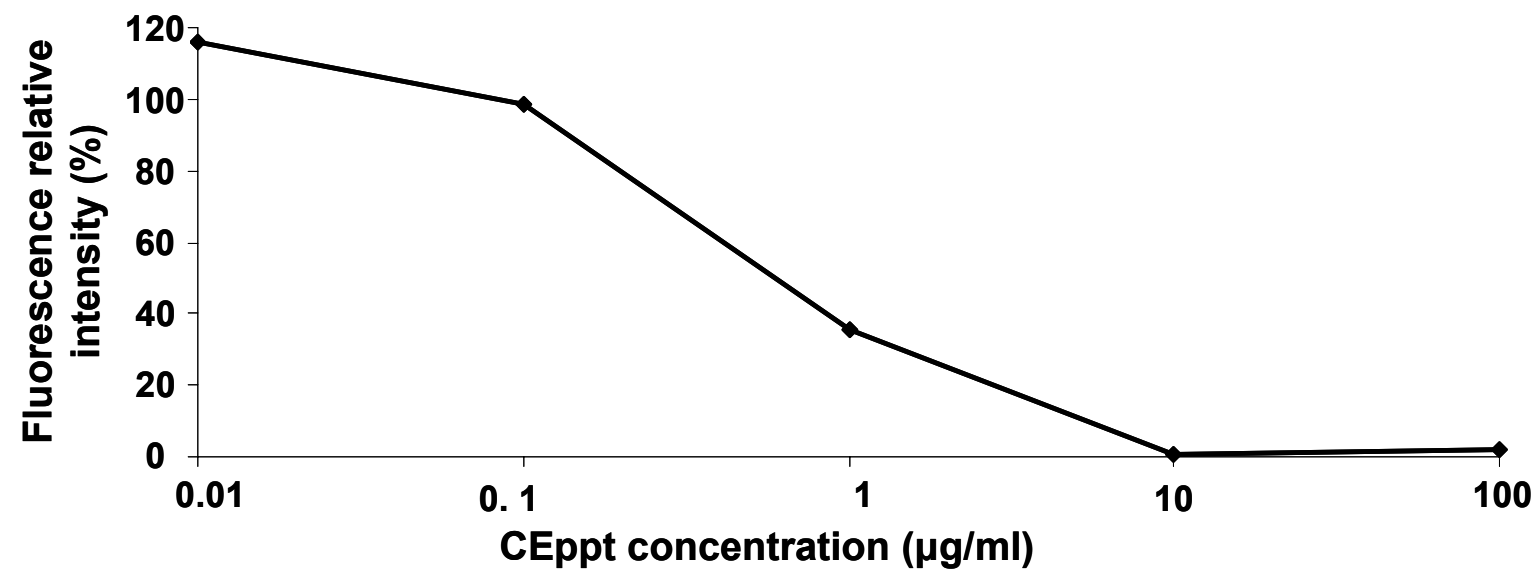

Supplement: Figure S2 — Concentration-dependent inhibition of Aβ40 fibrillogenesis. CEppt was added at different concentrations to a fixed amount of 20 µg/ml Aβ40. After incubation for 264 hours at 37°C, ThT fluorescence was monitored at an emission wavelength of 480 nm (excitation at 450 nm). (PDF) [file pone.0016564.s002.pdf]

A

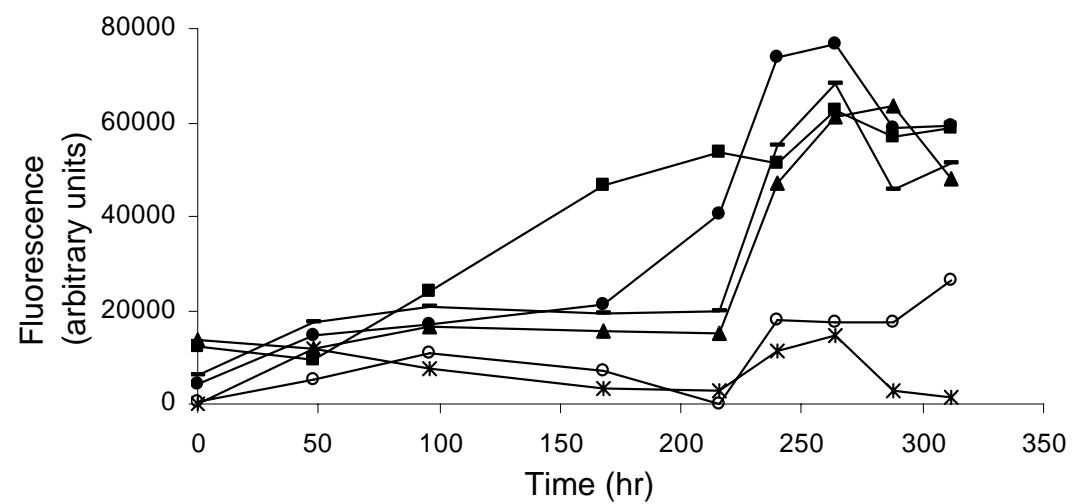

B

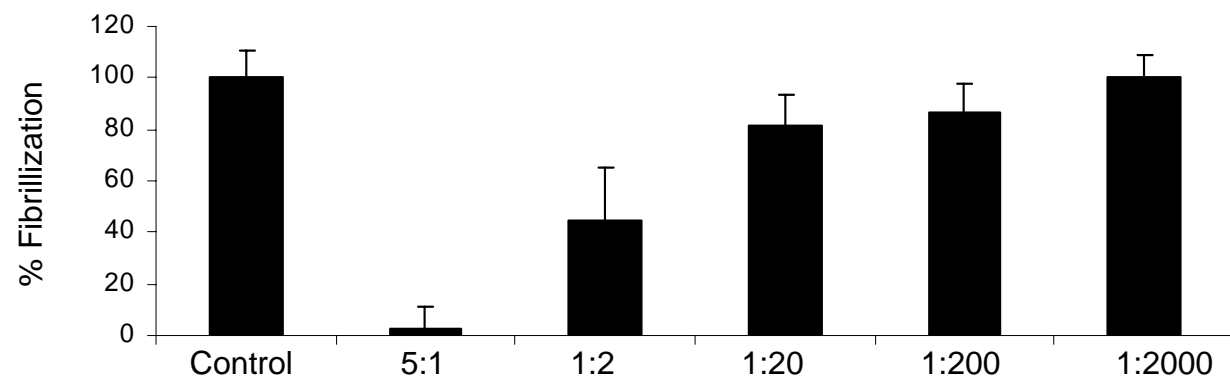

Supplement: Figure S3 — Inhibition of Aβ42 assemblies in vitro. (A) The kinetics of Aβ42 (20 µg/ml) fibril formation in the absence or presence of CEppt as assessed by the Thioflavin-T binding assay over the course of 312 hours. Concentrations are expressed as CEppt:Aβ42 concentration ratio (w/w). Control - Aβ42 only (▪); 5∶1 (*); 1∶2 (o); 1∶20 (▴); 1∶200 (-); 1∶2000 (•). (B) Endpoint of ThT analysis measurement T = 312 hours. Concentrations are expressed as CEppt: Aβ42 concentration ratio (w/w), control is Aβ42 (20 µg/ml). (PDF) [file pone.0016564.s003.pdf]

**A**

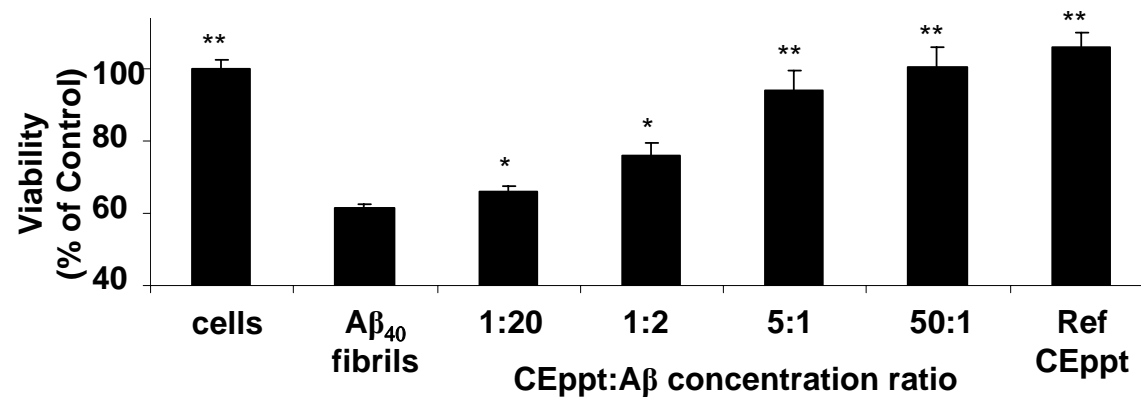

**B**

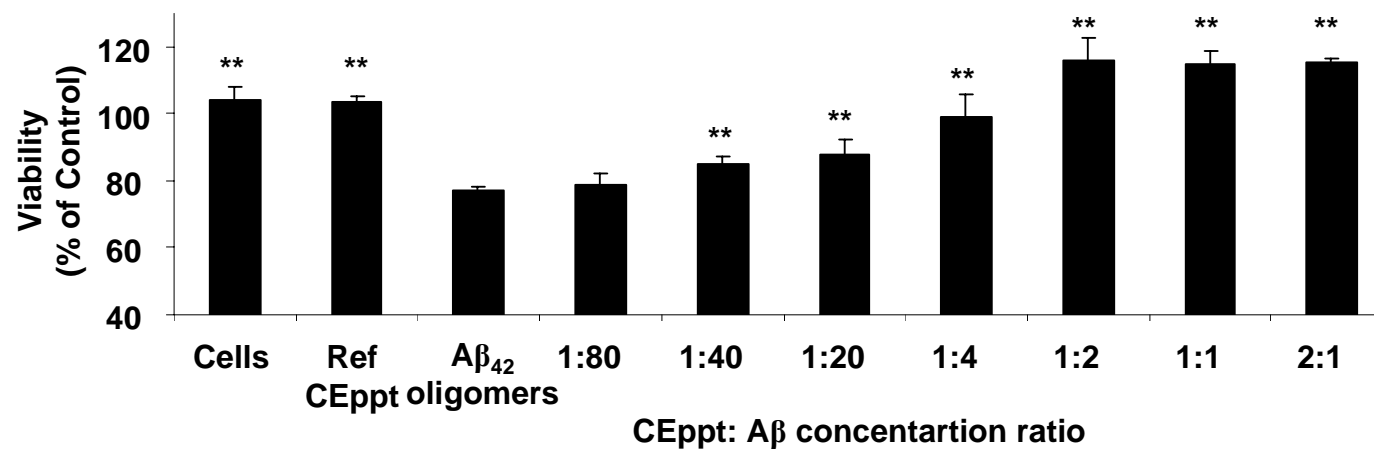

Supplement: Figure S4 — CEppt alleviates toxic effects of Aβ oligomers and fibrils in cell assay. (A) Samples of Aβ40 (20 µg/ml) with or without various concentrations of CEppt were incubated for 24 hours with PC12 cells culture. To exclude any toxic effect of CEppt, CEppt alone (1 mg/ml) was pre-incubated for 24 hours with PC12 cells (ref CEppt). Cells viability was determined using MTT viability assay. (B) Samples of Aβ42 oligomers (0.6 mg/ml) with or without various concentrations of CEppt were incubated for 24 hours with PC12 cells culture. To exclude any toxic effect of CEppt, CEppt alone at the highest concentration was pre-incubated for 24 hours with PC12 cells (ref CEppt). Cells viability was determined using MTT viability assay. * Pv< 0.05, ** Pv<0.005. (PDF) [file pone.0016564.s004.pdf]
